# Impact of the Soak and the Malt on the Physicochemical Properties of the Sorghum Starches

**DOI:** 10.3390/ijms11083002

**Published:** 2010-08-16

**Authors:** Irakoze Pierre Claver, Haihua Zhang, Qin Li, Kexue Zhu, Huiming Zhou

**Affiliations:** 1 State Key Laboratory of Food Science and Technology, Jiangnan University, Lihu Road 1800, Wuxi 214122, China; E-Mails: irakozefr@yahoo.fr (I.P.C.); zhanghaihua2008@yahoo.com.cn (H.Z.); liqin200809.funny@163.com (Q.L.); kxzhu@jiangnan.edu.cn (K.Z.); 2 School of Food Science and Technology, Jiangnan University, 1800 Lihu, Wuxi 214122, Jiangsu Province, China; 3 Université du Burundi, Institut Supérieur d’Agriculture, Département de TIAA. BP. 35 Gitega, Burundi

**Keywords:** sorghum, malt, soak, starch, X-ray diffractograms, RVA, DSC, crystallinity

## Abstract

Starches were isolated from soaked and malted sorghum and studied to understand their physicochemical and functional properties. The swelling power (SP) and the water solubility index (WSI) of both starches were nearly similar at temperatures below 50 °C, but at more than 50 °C, the starch isolated from malted sorghum showed lower SP and high WSI than those isolated from raw and soaked sorghum. The pasting properties of starches determined by rapid visco-analyzer (RVA) showed that malted sorghum starch had a lower viscosity peak value (86 BU/RVU) than raw sorghum starch (454 BU/RVU). For both sorghum, X-ray diffractograms exhibited an A-type diffraction pattern, typical of cereal starches and the relative degrees of crystallinity ranged from 9.62 to 15.50%. Differential scanning calorimetry (DSC) revealed that raw sorghum starch showed an endotherm with a peak temperature (Tp) at 78.06 °C and gelatinization enthalpies of 2.83 J/g whereas five-day malted sorghum starch had a Tp at 47.22 °C and gelatinization enthalpies of 2.06 J/g. Storage modulus (G′) and loss modulus (G″) of all starch suspensions increased steeply to a maximum at 70 °C and then decreased with continuous heating. The structural analysis of malted sorghum starch showed porosity on the granule’s surface susceptible to the amylolysis. The results showed that physicochemical and functional properties of sorghum starches are influenced by soaking and malting methods.

## 1. Introduction

As more than 500 million people in developing countries depend on sorghum as the main staple food, relevant scientific information generated for this crop can certainly play a key role in food development [1]. Among carbohydrate polymers, starch is currently enjoying attention owing to its usefulness in different food products. Sorghum, like other cereals, is rich in starch–a major storage form for carbohydrates–which makes up about 60–80% of normal kernels and has excellent potential for industrial applications [2,3].

Starch is used in a variety of food products as a raw material or food additive, and has an important role as a thickener, bulking agent, gelling agent, water absorbent and is used also in foods with varying moisture contents such as puddings, cookies, or drinks. Some of the starch derivatives are being increasingly used as fat substitutes [4,5].

Starch modification, which involves the alteration of the physical, chemical and functional characteristics, can be used to tailor starch to specific food applications.

Chemical or enzymatic modification involves the introduction of functional groups into the starch molecule, resulting in markedly altered physicochemical properties. Such modification profoundly alters their gelatinization, pasting and retrogradation behavior [6]. It was observed that the chemical treatment was effective in starch modification, but also exhibited side effects. Soaking and malting is simple, safe and can also improve the quality of sorghum flour in terms of reducing the anti-nutrients. However data on soaked and malted sorghum starch properties are limited and little is known about the functional and physicochemical properties of soaked and malted sorghum starch. The characterization of sorghum starches is particularly relevant before considering their industrial utilization which is important to preserve the local sorghum varieties and enhance the sorghum cultivation that contribute to the socioeconomic development. Also, to our knowledge, wood ash extract during soaking has not yet been conducted. The wood ash is an alkaline material (pH = 10) that is used to improve the organoleptic quality of malted sorghum flour. Soaked and malted sorghum starch is essential for food formulation and could also show potential uses in breweries as well for the production of stiff and weaning food with low paste viscosity and high energy density.

In light of the above considerations, this paper focused on physicochemical and functional properties of sorghum starch such as, swelling power, water solubility, crystallinity, starch granular structure and pasting properties in order to better understand the effect of soaking and malting on sorghum starch modification, since the endogenous enzymes of sorghum are susceptible to modification of the molecular structure of sorghum starch during soaking and malting. Keeping this in view, the present work was undertaken to evaluate the sorghum starch modification by endogenous enzymes during soaking and malting, which could suggest the possibility of using sorghum starch as a potential raw material for food industry.

## 2. Materials and Methods

### 2.1. Materials

Sorghum (*Sorghum bicolor (L.*) *Moench*) was grown in Shandong, a coastal province East of China and known to have an average temperature of 0 °C in January and 28 °C in July. The average annual rainfall is about 500 mm, most of which falls in the summer. Red sorghum was obtained from the 2007 and 2008 harvest. The length/breadth ratio of sorghum kernel was 1.12/1.23 and the density (g/L) was 691.40. The average weight of 1000 kernels was 26.80 g.

All the chemicals used were of analytical grade and purchased from Sinopharm Chemicals Reagent Company (SCRC), Shanghai, China.

### 2.2. Malting Procedure

After removing chaff, sorghum grains (2000 g) were thoroughly cleaned by washing with tap water and then soaked in 0.20 ppm wood ash water for 24 h at 25 °C, with the soaking water changed at 8 h intervals. After soaking, the grains were evenly spread on jute bags and covered with the same material, in a secluded and dark area, and allowed to malt. The temperature of malting kernels was 25 °C and growth was terminated at 3 and 5 days by kilning in a forced air oven at 40 °C for 24 h. The withered rootlets were gently brushed off and dried grains were milled using a bench-top attrition mill (Dade, DFT-600, 25,000 rpm, Zhejiang Linda Mechanic Ltd Co, China). The resultant flour was sieved into a particle size of 212 μm. The flour was then packaged in a low density polyethylene bag and stored using plastic air tight containers at 4 °C for later use.

### 2.3. Starch Isolation

Starch extraction was performed according to the method described by Choi with some modifications [7]. Briefly, the flour sample was immersed in 0.25% aqueous NaOH solution and kept at 5 °C for 24 h. Before stepwise filtration through 60 (250 μm), 100 (149 μm), 270 (53 μm) and 400 (37 μm) mesh sieves, the slurry was blended (BL530, Kenwood, Japan) for about 3 min. Double deionized water was used to wash the slurry until no white starch was washed out. The filtrate was then centrifuged by freezing centrifugation (ZOPR-52D, Hitachi Koki Co, Japan) at 10,000 rpm for 25 min. The supernatant was discarded, and the top yellow protein layer was also removed. The lower starch layer was resuspended in double deionised water, and centrifuged as above, and this procedure was repeated twice. The isolated starch was lyophilized (ACPA1-4, Christ Co. Germany) and ground to pass through a mesh 70 (212 μm) sieve.

### 2.4. Swelling Power and Water Solubility Index

The estimation of SP and WSI was done according to the method of Tang with some modifications [8]. A suspension of 0.10 g of starch and 6 mL of distilled water was heated in a water bath in the range of 30–80 °C for 30 min. The suspension was then cooled rapidly at room temperature and centrifuged (8,000 rpm, 20 min). WSI was reported as the ratio of dry mater supernatant to dry starch sample whereas SP was reported as the ratio of swelling starch granules sediment to dry starch. Three replicate samples were used in this determination.

### 2.5. Pasting Properties

The pasting properties were determined by RVA (Brabender, Duisburg, Germany). Sorghum starch slurry (10 g), dispersed in 100 mL of deionized water was directly placed into a stainless steel measuring bowl and then heated from 30 to 95 °C, held for 30 min at 95 °C and then cooled to 50 °C and held 30 min at this temperature. Heating and cooling rates were 3 °C/min. Pasting temperature (PT), Peak viscosity (PV), hot paste viscosity (HPV), Viscosity at the end of holding at 95 °C, final viscosity at 50 °C or cool paste viscosity (CPV), were recorded.

### 2.6. X-ray Diffraction Patterns of Starch Granules and Relative Crystallinity

X-ray patterns of all samples were obtained with copper (nickel foil filtered) Kalpha radiation using powder X-ray diffractometer (D8S AXS, Germany, Bruker). The operation setting for the diffractometry was 40 kV and 40 mA. Data were collected from 1–70° 2Th (Th is theta, the angle of diffraction) with a step width of 10° and step time of 25 s. Values of 2Th for each identifiable peak on the diffractograms were estimated, and d-spacing were calculated using Bragg’s law. The relative degree of crystallinity was estimated by determining the ratio of the area, in counts, under major diffraction peaks to the total area under the curve between 8 and 35° 2Th according to the Hayakawa method [9]. The areas were determined by weighing the two sections. The areas were calculated by comparison of their weights with the weight of known areas prepared using the same paper.

### 2.7. Thermal Properties

A DSC (Pyris-1, Perkin–Elmer) was employed to conduct thermal analyses for individual native sorghum starches and their mixtures. Approximately 1.00 mg of starch samples were loaded into aluminum sample pans. Deionized water was added by micropipette to achieve a mixture with a required water content of 70% (w/w) in starch. The sample pans were sealed and equilibrated at room temperature for 1 day before analysis. The samples were heated at 10 °C/min over a temperature range of 25–100 °C using an empty pan as a reference. Parameters such as onset (To), peak (Tp), conclusion (Tc) temperature and enthalpy (ΔH) were automatically recorded using Seiko software.

### 2.8. Viscoelastic Properties

Dynamic rheological properties during heating and cooling cycles of the starch suspensions were determined using an AR 100 Rheometer (TA Instruments, UK) with parallel-plate geometry of 40 mm diameter and gap geometry of 1000 μm. Starches were loaded onto the ram of the rheometer and covered with a thin layer of low-density silicon oil to minimize evaporation and rheological properties were described in terms of storage (G′), loss modulus (G″). Starch suspensions were scanned during heating from 20 to 95 °C and respectively during cooling from 95 to 20 °C at a rate of 5 °C/min with a strain and frequency set at 2% and 5 rad/s. All rheological measurements were conducted in duplicate. The software supplied by the equipment manufacturer was used to examine the suitability of common rheological models with reference to the standard errors.

### 2.9. Morphological Properties

Scanning electron micrograph (SEM) studies of the starch were carried out using an Electro scan model Quanta 200 environmental scanning microscope (Fei Company, Netherlands). The samples were coated with a thin layer of gold in a Fine Coat Ion Sputter JFC 1100 before obtaining the micrographs. Coating surface and cross section were examined using an accelerating voltage of 10 kV. The image was captured using 9.0 mm Ricoh Camera of 160 × mga.

### 2.10. Statistical Analyses

All analyses were carried out in triplicates and data were presented as mean ± SD. Analysis of variance (ANOVA) was carried out using SAS package (SAS Institute, Cary, NC). Comparisons between means were done using a Duncan’s multiple-range test with a probability of *P* < 0.05.

## 3. Results and Discussion

### 3.1. Swelling Power and Water Solubility Index of Sorghum Starches

The SP and WSI at different temperatures ranging between 55 and 95 °C are presented in Table 1. The SP and WSI of starches increased with increasing temperature. At temperatures lower than 50 °C the SP of all starches were similar, whereas above this temperature, the SP of malted sorghum starch was lower than that of raw sorghum starch. At higher temperatures (>50 °C), a sudden increase in SP was observed for all starches. Starch swelling power is attributed to the strength and character of the micellar network within the starch granule. As the temperature increased, the starch vibrated more vigorously, breaking intermolecular bonds and allowing hydrogen-bonding sites to engage more water molecules. SP and WSI provide evidence of the magnitude of the interaction between starch chains within both the amorphous and crystalline domains [10]. The WSI variation profiles for both starches were similar. It leveled off at temperatures between 50 and 80 °C and became high for malted sorghum starch than that of raw and soaked sorghum. Starch from grain malted for five days showed the highest WSI (16.53%) at 70 °C whereas raw material starch showed the lowest WSI (10.54%) at the same temperature. This is to be expected because the reserves of nutrients like starch and protein are respectively degraded by internal enzyme to soluble sugars and amino acids to meet the seedling requirements in the germination process. In addition, amylose is more soluble than amylopectin and the behavior further suggests that the malted sorghum starch had considerably larger amounts of amylose than raw sorghum starches. SP of starches from different malted sorghum were found in the range of 18.54 and 24.53.57 g/g, being the highest for raw grain and lowest for sorghum grain malted for three or five days.

Subrahmanyam and Hoseney reported SP between 13.8 and 15.2 g/g and WSI between 17.4% and 22.5% for starches isolated from seven US sorghum cultivars [11]. Olayinka reported SP of 8.79 g/g and WSI of 5% for Nigerian sorghum starch [12]. In general genetic and environmental factors as well as starch isolation procedure are attributed to the differences in the SP and WSI of sorghum starches.

### 3.2. Pasting Properties of Sorghum Starches

**Figure 1:**
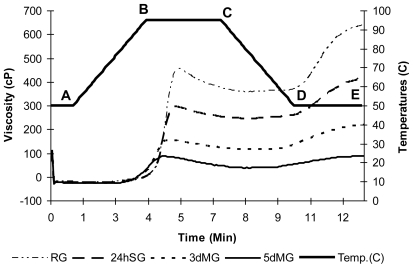
RVA-viscograms of soaked and malted sorghum starches. RG: raw grain; 24hSG: 24 hours soaked grain; 3dMG: 3 days malted grain; 5dMG: 5 days malted grain. A–B: heating period, B–C: holding period, C–D: cooling period, D–E: final holding period.

The pasting properties of sorghum starches using RVA are presented in Figure 1. The raw sorghum starches took longer to gelatinize than the malted ones and had a significantly higher value for peak viscosity (454 BU/RVU) and final viscosity (637 BU/RVU) than malted sorghum starches. Malting significantly lowered the values of peak viscosity (157 BU/RVU) and cooking viscosity (114 BU/RVU) for 3 days malting while 5 days malting showed 86 BU/RVU of peak viscosity and 37 BU/RVU of cooking viscosity. Before the malting procedure of sorghum grain, the viscosity was relatively high and upon malting it dropped for all the samples. During the hold period at 95 °C, the sample is subjected to a period of constant high temperature and shear stress. This will further disrupt the granules, and amylose molecules will continue to leach out into solution. This period is commonly accompanied by a breakdown in viscosity, sometimes called trough. The rate of viscosity reduction depends on the temperature and degree of shear applied to the mixture, and the nature of the material itself. The ability of the granules to withstand the heating and shear is an important factor for many food-processing situations [13].

As the mixture is subsequently cooled, re-association between starch molecules occurs. In sufficient concentration this causes the formation of a gel, and viscosity will normally increase. This phase of the pasting curve is commonly referred to as the setback region, and involves retrogradation, or re-ordering, of the starch molecules. The increase in viscosity occurs not only due to the simple kinetic effect of cooling on viscosity, but also due to the re-association of starch molecules. Final viscosity is the most commonly used parameter to define a particular sample’s quality, as it indicates the ability of the material to form a viscous paste (stickiness) or gel after cooking and cooling.

Setback is sometimes measured as the difference between final viscosity and peak viscosity, which has been related to the firmness and the amylose content. Breakdown is defined as subtracting the viscosity at the trough from the final viscosity [14]. The reduction in viscosity of malted sorghum can be attributed to starch degradation caused by the action of *alpha* and *beta*-amylase developed during the malting process. Amylose is more susceptible to degradation than amylopectin, and malted sorghum is less viscous than normal sorghum [13].

### 3.3. X-ray Diffraction Pattern and Relative Crystallinity of Sorghum Starches

**Figure 2:**
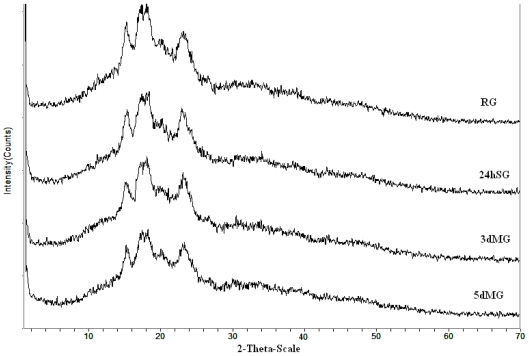
X-ray diffraction patterns of sorghum starch obtained after soaking and malting. RG: raw grain; 24hSG: 24 hours soaked grain; 3dMG: 3 days malted grain; 5dMG: 5 days malted grain.

The X-ray diffractograms of sorghum granules are shown in Figure 2. Strong peaks were observed at 2Th values of 15.206, 17.404 and 23.154 Å, corresponding to *d-*spacing (inter planar distances) of 5.8219, 5.0911 and 3.8382 Å, respectively. This pattern closely matches reported values for A-types cereal starches. The same peaks were found for sorghum cultivated in the Sahara of Algeria [15]. The X-ray diffraction pattern of the RG gave also a peaks around 2Th = 20, that can be attributed to different crystalline structures which are typical patterns of V-amylose peak (inclusion complex of amylase with lipid) and should be distinguished from that of malted sorghum starches.

The crystallinity degrees were 9.62, 11.45, 13.32 and 15.51%, for 5dMG, 3dMG, SG and RG respectively. Percent crystallinity values for native starch correlated well with enthalpy of gelatinization calculated on a dry basis of starch [16]. Five days malted sorghum starch exhibited slight lower crystallinity compare to raw sorghum starches and the degree of crystallinity is reported to be one of the several factors that determine starch digestibility [17]. These major crystalline changes diffraction pattern indicated the impact of soaking and malting on amylose organization. In other words, soaking and malting remove amylopectin-based ordered structure and generates amylose-based ordered structure. It is interesting to note that endogenous enzymes after malting did not lead to a fundamental change in the overall pattern that contains both V- and B-type starch diffraction.

The degree of crystallinity perfection according to Tester and Morison is impacted by the molecular structure of amylopectin such us unit chain length, extent of branching, molecular weight, and polydispersity however, no experimental proof exists to exclude the presence of amylose in the crystalline regions [18,19].

### 3.4. Thermal Properties of Sorghum Starches

Thermal properties of the sorghum starches are shown in Table 2. T_o_ of sorghum starches were ranged from 36.98 to 71.74 °C and the high temperature was found with the starches derived from RG while the lowest was observed with 5dMG. Gelatinized sorghum starches were ranged from 47.22 to 78.06 °C for T_p_ and 37.48 to 75.03 °C for T_c_ and the highest values were found for starches derived from RG. The enthalpy of starch gelatinization varied from 2.06 to 2.83 J/g and was found decreasing with the time of malting. Significant positive correlations of ΔH with T_o_ (r = 0.939, *P* ≤ 0.005), T_p_ (r = 0.944, P ≤ 0.005) and T_c_ (r = 0.938, P ≤ 0.05) were observed and the peak height index (PHI) was ranged from 0.12 (24hSG) to 0.43 (5dMG). The decreases of ΔH values for malted sorghum starch are evidence of the low amount of crystalline fraction of the substance [20]. Sang observed T_o_, T_p_, T_c_ of 67.9, 70.7, 75.7 °C respectively and ΔH of 13.2 J/g for common sorghum starch [21]. Beta observed 67.4 °C an average of T_p_ and ΔH of 7.45 J/g for ten Zimbabwean sorghum starches [22]. Gaffa reported a T_c_ of 90 °C and ΔH of 13.7 J/g for Nigerian sorghum starch [23].

The difference in gelatinization ranges R (R = gelatinization range defined as difference between T_c_ and T_o_ among the starches) was significant and ranged between 0.5 °C for 5dMG and 3.29 °C for RG. The peak width (T_c_-T_o_) generally broadened and the enthalpy values (ΔH) decreased upon hydrolysis. The differences in the R values amongst the starches from different cultivars may be attributed to the presence of crystalline regions within a starch granule composed of small crystallites having slightly different crystal strength. Starch gelatinization for Korean waxy sorghum was reported to have 73.2 °C [7]. Compared to results obtained by Singh [10] for starches of other botanical sources as potato, corn, rice and wheat, local sorghum starch showed higher onset and peak temperature and lower gelatinization enthalpy. High temperature gelatinization can be an indication of the higher stability of starch crystallites in starch molecules. Starch gelatinization temperature is influenced by many factors; in particular the lengths of the various chains in the amylopectin molecule with gelatinization temperature increasing with longer chain length [24]. The gelatinization enthalpy depends on number of factors such as crystallinity intermolecular bonding, and it also depends on genetic and environmental factors. Five days malted sorghum starch significantly showed a low gelatinization temperature with a low enthalpy than raw grain, soaked and three-days malted sorghum starch. Time of malting was a significant factor in determining the ability of sorghum starches to gelatinize at low temperature. Starch gelatinization results in its rapid breakdown by amylases, there is a positive correlation in starch hydrolysis and the temperature of gelatinization.

Since gelatinization rates are related to starch composition and structure, the extent of starch modification during grain malting may influence rates of gelatinization. Therefore, the decrease in gelatinization rates for starches from the malted sorghum starch may suggest improvements in starch degradability possibly due to differences in the degree of modification of starch structure and composition.

### 3.5. Rheological Properties of Sorghum Starches

#### 3.5.1. During Heating

**Figure 3:**
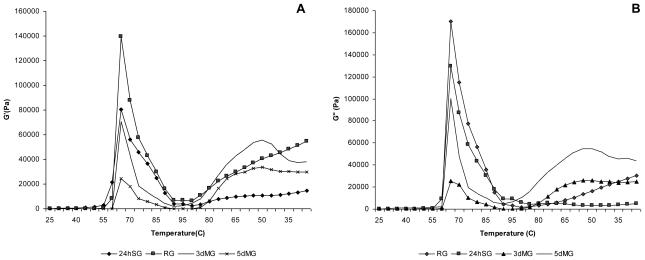
Development of G′ and G″ modulus in soaked and malted sorghum starches suspension during heating from 25 to 95 °C and cooling from 95 to 25 °C. RG: raw grain; 24hSG: 24 hours soaked grain; 3dMG: 3 days malted grain; 5dMG: 5 days malted grain.

As illustrated in Figure 3, storage modulus (G′) and loss modulus (G″) of all starch suspensions increased steeply at maximum of 70–73 °C and then decreased with continuous heating. More detailed examination indicated that the temperature (T_G′max_) at which the G′ of RG and 24hSG reached the maximum value (71 °C) and was consistently higher than that of 3dMG and 5dMG. Svegmark and Hermansson reported that, during the heating cycle, the difference in the G′, G″ should be due to the difference in starch granule swelling and leaching of amylose chains [25]. This contributes to the formation of a composite network of solvated materials supporting partially disintegrated starch granules [26]. Further heating results in extended rupture and disintegration of starch granules with melting of crystallites and weakening of interchain interactions due to the increasing of molecular mobility, hence manifesting a reduction of G′ and G″ [27]. Regarding the process of malting, viscoelastic functions were reduced considerably with increasing dose of the treatment that affected adversely the integrity of the starch granule.

#### 3.5.2. Controlled Cooling

At temperatures between 90 and 70 °C, traces of G′ and G″ remained relatively flat, consistent with what has been reported for cultivars of rice and apple [28]. All samples of malted sorghum exhibited low peaks in shear modulus, in contrast, the corresponding traces for RG and 24hSG were relatively steep forming a sigmoidal pattern of modulus development. These results indicated that the effect of malting influenced the starch composition and starches from malted grain were not capable of forming cohesive networks. It has been stated that starch with relatively low amylose content exhibits a modest increase in storage modulus of networks upon cooling [29]. Upon cooling and storage, the RS starch exhibited a greater development of the elastic (G′), indicating the formation of strong three*Int.* dimensional networks [30]. The poor gelation and retrogradation properties of starches may be associated with the relatively short length of the linear chains in amylopectin of these starches [31]. It appears that the partial depolymerization of starch polymers increased the mobility and diffusion of chains. Lauro [32] reported that stiffness of amylopectin gels decreased substantially with increasing level of amylopectin degradation. It appears that even a small fragmentation of amylopectin polymers during partial enzymatic or acidic hydrolysis completely diminished their potential for gelation and retrogradation. This behavior may be particularly useful in certain food applications.

### 3.6. Scanning Electron Micrograph

**Figure 4:**
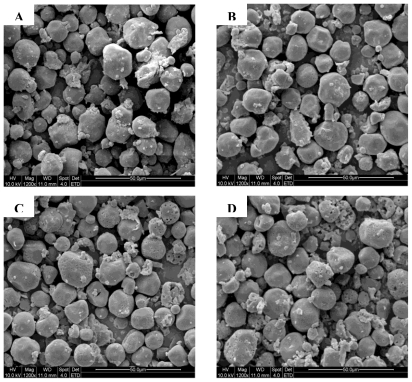
Scanning electron micrographs of starch from soaked and malted sorghum. A: raw grain; B: 24 h of soaked sorghum grain; C: 3 days malted grain; D: 5 days malted grain.

When viewed with SEM, sorghum starch differed in their internal structures. The partially degraded starch granules were examined using SEM, to study the effect of internal enzyme on the morphology of the starch granules during the period of soaking and malting. From the SEM photographs, it was evident that hydrolysis of starch didn’t occur uniformly at the beginning of the process. Some areas were much more susceptible to be attacked than others (Figure 4A). When sorghum grain was soaked for 24 h, starch was hydrolyzed and heterogeneity was observed in the starch granule degradation; most granules remained intact or were only slightly modified (Figure 4B). Sorghum malted for three days showed the appearance of large pin holes (Figure 4C). Enzymes caused surface alterations and degraded the external part of the granule during the period of malting. The internal enzyme acts by first attacking the surface and forming pores on the surface [33] and when endo-corrosion occurred, the internal part of the granule was corroded through small pores by which enzymes penetrated the granule. After five days of malting, small granules were more rapidly hydrolyzed because of a large available surface area (Figure 4D).

Generally, enzymes can erode the entire granule surface or digest channels from selected points on the surface towards the center of the granule [34]. *Alpha*-amylases are the endo-acting enzymes that randomly attack the internal alpha-1,4-*D*-glucosidic linkages of starch except those near the branch points that contain alpha-1,6-*D*-glucosidic linkages [35]. The presence of pores, channels and cavities increases the surface area, which is potentially available for chemical and enzymatic reactions. Fannon suggested that pores may be the site of initial enzyme attack, openings that allow enzyme molecules direct access to the granule interior [33]. Enzyme molecules influence the starch granule surface in different ways. Five patterns of enzymes attack have been identified: pin-holes, sponge-like erosion, numerous medium-sized holes, distinct loci leading to single holes in individual granules, and surface erosion (Evers, 1979). Generally, enzymes can erode the entire granule surface or sections of it (exo-corrosion) or digest channels from selected points on the surface towards the center of the granule (endo-corrosion). Enzymatic reaction consists of a few stages: diffusion towards the surface of solid phase, adsorption, and finally catalytic reaction. This process is divided into several phases and after random adsorption of the enzymes at the granule surface, the process of hydrolysis starts. Then, the hydrolysis proceeds rapidly, forming pores that facilitate free diffusion of the enzymes into the granule. This leads to the formation of channels, which finally reach the center of the granule. The pits seen in malted starch are directly the result of the partial starch hydrolysis by internal enzymes during malting.

## 4. Conclusions

The results indicated significant changes in terms of functional properties of sorghum starches obtained after soaking and malting. Functional properties related to starch structures provided information about the structural changes that occurred during the soaking and malting process. Starches isolated from malted sorghum showed lower SP and higher WSI than those isolated from raw and soaked sorghum. The pasting properties of starches determined by RVA showed that malted sorghum starch had a lower viscosity peak value than raw sorghum starch. The changes observed in physicochemical, morphological, thermal and rheological properties of sorghum starches after malting provided a crucial basis for understanding starch modification mechanisms with the potential applications at industrial scale. The raw sorghum starches exhibited very interesting functional properties suitable to be utilized in food products as thickening and gelling agent for their higher values of viscosity. Soaked and malted sorghum starches were essential for food formulation and could also show potential uses in brewery as well for the production of stiff and weaning food with low paste viscosity. Functional characterization showed that sorghum starches are potential functional food ingredient.

Sorghum starches thus have excellent applications for future product development by virtue of their functional properties.

## Figures and Tables

**Table 1:** Swelling power and water solubility index patterns of sorghum starches.

Temperature (°C)	RG	24hSG	3dMG	5dMG
Swelling power				
50	6.25 ± 0.07a	5.65 ± 0.41a	5.76 ± 1.10a	4.65 ± 0.57a
60	18.68 ± 0.19a	16.45 ± 0.74b	14.23 ± 0.04c	9.34 ± 0.90d
70	24.53 ± 0.77a	21.01 ± 0.45b	19.62 ± 1.52b	18.54 ± 0.90b
80	22.43 ± 0.36a	20.01 ± 0.2.6ab	18.68 ± 1.14bc	17.01 ± 0.93c
Water solubility index				
50	4.65 ± 0.74a	5.65 ± 0.59a	5.76 ± 0.77a	6.25 ± 1.03a
60	9.34 ± 0.32b	10.45 ± 0.31ab	11.65 ± 1.89ab	12.68 ± 0.63a
70	10.54 ± 0.49c	12.54 ± 0.84bc	13.56 ± 0.74b	16.53 ± 0.53a
80	9.42 ± 0.77c	11.45 ± 0.74b	12.78 ± 0.28ab	14.43 ± 0.63a

RG: raw grain; 24hSG: 24 h soaked grain; 3dMG: 3 days malted grain; 5dMG: 5 days malted grain. All values are means of three replicates. Values with same letters (a, b, c, d within rows) are not significantly different at *P* < 0.05.

**Table 2:** Gelatinization characteristics of soaked and malted sorghum starch.

Sample	RG	24hMG	3MG	5MG
T_o_ (^o^C)	71.74 ± 0.97a	41.04 ± 2.43b	38.78 ± 0.79b	36.98 ± 1.58b
T_p_ (^o^C)	78.06 ± 1.93a	51.44 ± 1.40b	49.21 ± 1.72b	47.22 ± 0.67b
T_c_ (^o^C)	75.03 ± 0.66a	42.13 ± 1.56bc	39.28 ± 2.09b	37.48 ± 1.49c
ΔH (J/g)	2.83 ± 0.32a	2.30 ± 0.28ab	2.36 ± 0.05c	2.06 ± 0.01b
R (^o^C)	3.29 ± 0.04a	1.09 ± 0.02b	0.50 ± 1.30c	0.50 ± 0.01c
PHI	0.43 ± 0.11a	0.24 ± 0.11ab	0.12 ± 0.00b	0.19 ± 0.04ab

T_o_, T_p_ and T_c_ indicate respectively the temperature of the onset, peak and conclusion of gelatinization; ΔH enthalpy of gelatinization; R = gelatinization range (T_c_ - T_o_), PHI = peak height index ΔH/(T_p_-T_o_); RG: raw grain; 24hMG: 24 h malted grain; 3dMG: 3 days malted grain; 5dMG: 5 days malted grain. All values are means of three replicates ± SD. Values with same letters (a,b,c within raw) are not significantly different at *P <* 0.05.

## Abbreviations

ANOVA: Analysis of variance
BU: Brabender units
CPV: Cool paste viscosity
DSC: Differential scanning calorimetry
HPV: Hot paste viscosity
MG: Malted grain
PHI: Peak height index
PT: Pasting temperature
PV: Peak viscosity
RG: Raw grain
RVA: Rapid visco analyzer
RVU: Rapid viscoanalyser units
SCRC: Sinopharm chemicals reagent company
SD: Standard deviation
SEM: Scanning electron micrograph
SG: Soaked grain
SP: Swelling power
T_c_: Conclusion temperature
T_d_: Denaturation temperature
Th: Theta, the angle of diffraction
T_o_: Onset temperature
T_p_: Peak temperature
WSI: Water solubility index
ΔH: Denaturation enthalpy
